# Evaluation of an Infection surveillance program in residential aged care facilities in Victoria, Australia

**DOI:** 10.1186/s12889-023-17482-x

**Published:** 2024-01-22

**Authors:** Eliza Watson, Arjun Rajkhowa, David Dunt, Ann Bull, Leon J Worth, Noleen Bennett

**Affiliations:** 1 Doherty Institute for Infection and Immunity, Victorian Healthcare Associated Infection Surveillance System (VICNISS) Coordinating Centre, 792 Elizabeth St, Melbourne, VIC 3000 Australia; 2 Department of Infectious Diseases, National Centre for Antimicrobial Stewardship, The University of Melbourne, Melbourne, VIC 3000 Australia; 3 The University of Melbourne, Melbourne, VIC 3000 Australia; 4 Department of Oncology, Department of Infectious Diseases, University of Melbourne Cancer, Peter MacCallum Cancer Centre, Melbourne, VIC 3000 Australia; 5 Department of Nursing, Melbourne School of Health Sciences, The University of Melbourne, Melbourne, VIC 3065 Australia

**Keywords:** Nursing home, Homes for the aged, Cross infection, Infection surveillance, Public health surveillance, Mixed methods

## Abstract

**Background:**

Infection surveillance is a key element of infection prevention and control activities in the aged care sector. In 2017, a standardised infection surveillance program was established for public residential aged care services in Victoria, Australia. This program will soon be expanded to a national level for all Australian residential aged care facilities. It has not been evaluated since its inception.

**Methods:**

The current study aimed to evaluate the Victorian Healthcare Associated Infection Surveillance System (VICNISS) Coordinating Centre Aged Care Infection Indicator Program (ACIIP), to understand its performance and functionality. A mixed methods evaluation was performed using the Updated Guidelines for Evaluating Public Health Surveillance Systems developed by the United States Centers for Disease Control and Prevention as a framework. VICNISS staff who coordinate and manage the ACIIP were invited to participate in interviews. Residential aged care staff who use the program were invited to participate in a survey. Document analysis was also performed.

**Results:**

Four VICNISS staff participated in the interviews and 38 aged care staff participated in the survey. The ACIIP is stable and able to be adapted quickly to changing definitions for infections. Users found the system relatively easy to use but have difficulties after the long intervals between data entry year on year. VICNISS staff provide expert guidance which benefits users. Users appreciated the benefit of participating and many use the data for improving local practice.

**Conclusions:**

The ACIIP is a usessful state-wide infection surveillance program for aged care. Further development of data validation, IT system capacity and models for education and user support will be required to support future scalability.

**Supplementary Information:**

The online version contains supplementary material available at 10.1186/s12889-023-17482-x.

## Introduction

Infection surveillance is a core component of effective infection prevention and control (IPC) programs. It is particularly important in residential aged care facilities (RACFs) because of the increased vulnerability of residents to infections. This increased vulnerability is due to a number of health and facility level concerns such as underlying chronic disease, impaired mental status, proximity of residents, and staff to resident ratios [1, 2]. Standardised surveillance ensures appropriate collection and reporting of results which can contribute to quality improvement programs and provides vital data for understanding the spread of infections [3].

In the state of Victoria, Australia, the Victorian Healthcare Associated Infection Surveillance System (VICNISS) Coordinating Centre was established in 2002 to coordinate standardised surveillance of healthcare associated infections (HAIs) in Victorian public acute care hospitals. In 2017, its scope of work was expanded to include the Victorian public sector residential aged care services (PSRACS). There are currently 179 PSRACS; most are located in regional and rural areas and range in size from 2 to 193 operational spaces [4]. PSRACS are funded by the Victorian State Government and are required to participate in the VICNISS Aged Care Infection Indicator (Surveillance) Program (ACIIP) [4].

In 2021, a National Health and Medical Research Council three-year research grant was awarded to three peak bodies - the Victorian Healthcare Associated Infection Surveillance System (VICNISS) Coordinating Centre, the National Centre for Antimicrobial Stewardship (NCAS) and the Registry for Senior Australians (ROSA) to oversee the development, implementation and evaluation of a national infection surveillance program for aged care (NISPAC). It is envisaged that this program will be accessible to all Australian RACFs and will include national access to an updated iteration of the ACIIP. This would require considerable expansion of the program and its capacity.

It is necessary that the ACIIP is reviewed to ensure that necessary improvements can be made and shortfalls are identified prior to development and scaling of a national program. Furthermore, ACIIP has not been evaluated since its inception in 2017 and it is important that programs of this size undergo evaluation to allow for continuous improvement and streamlining. In this study we utilised an international framework to perform a comprehensive evaluation of the ACIIP from the perspective of program users and VICNISS staff.

## Methods

A mixed-methods, multi-site evaluation study was conducted. The framework for evaluation was adapted from the Updated Guidelines for Evaluating Public Health Surveillance Systems developed by the United States Centers for Disease Control and Prevention (CDC) [5]. These guidelines were developed to ensure that surveillance systems are functioning effectively and outline several tasks to be conducted for evaluating surveillance systems. The evaluation of the VICNISS ACIIP involved (1) describing the program and its components and (2) an assessment of eight program attributes (Table 1). Evidence was generated through consultation with stakeholders via surveys and interviews, document analysis and systems review.

**Table 1:** Attributes from the CDC Updated Guidelines for Evaluating Public Health Surveillance Systems Program Evaluation that were used for the evaluation of VICNISS ACIIP [5]

Attribute	Definition
**Simplicity**	The ease of operation of the system and its integration with existing systems.
**Flexibility**	The ability of the system to adapt to changing information needs and/or operating conditions without significant changes in time, staff contribution or funding.
**Data quality**	The accuracy, completeness, and reliability of data captured.
**Acceptability**	The willingness of users to participate in the surveillance system.
**Representativeness**	The coverage of hospital reporting by geographical location and sector.
**Timeliness**	The timely entry, cleaning, analysis, and reporting of data by all users.
**Stability**	The reliability to maintain confidentiality and perform without failure, including during adaptation.
**Usefulness**	The ability of the system to achieve its objectives.

### Data collection

#### Document analysis

Two researchers (EW, NB) conducted analysis of relevant ACIIP documents including data collection forms, module protocols, issue logs, and support materials such as case studies and recorded webinars.

### Interviews

VICNISS staff involved in the coordination and management of ACIIP were approached to participate in semi-structured interviews. Interviews were conducted by one researcher (EW) via Zoom between 5th and 23rd of May 2022. Interview questions were mapped to the CDC evaluation attributes (Table 1) and were tailored to the staff members’ role in relation to ACIIP.

## Survey

Purposive sampling was used to recruit RACF staff registered with VICNISS and who had participated in the ACIIP in 2020 and/or 2021 on behalf of their facility. Invitations to participate in a 45-item survey conducted using Research Electronic Data Capture were sent via email (REDCap) [6, 7]. Twenty-seven questions focused on demographics, and infection and antimicrobial use surveillance in their PSRAC. The remaining 18 questions focussed on evaluation of the VICNISS ACIIP with questions mapped to the CDC evaluation attributes which can be assessed by PSRACS staff – simplicity, data quality, acceptability, timeliness and usefulness (Table 1). Survey questions included 5-point likert scale, multiple choice and open-ended options for comments.

Data from eligible staff who completed the VICNISS ACIIP evaluation section of the survey are presented.

### Data analysis

#### Qualitative data

Interviews were recorded, transcribed and uploaded to NVivo version 12 (QSR International) [8] along with the open-ended survey responses. Deductive coding was used to map data to the CDC evaluation attributes. Two researchers (EW and AR) independently coded the interview transcripts and discussed discrepancies. One researcher (EW) coded the survey responses.

### Quantitative

Data from the survey were analysed in STATA/SE 14.2 [9]. Frequencies of responses were calculated for each question.

### Triangulation

Interview, survey and document analysis data were analysed separately, with triangulation applied during interpretation of the results to determine whether the findings were convergent, dissonant or complimentary [10].

## Results

### Participants

Four VICNISS staff were interviewed: two IPC consultants, one manager and one IT officer. Interviews ranged from 14 to 37 min.

Fifty-seven staff from Victorian PSRACS participated in the survey; of these staff, 38 responded to the ACIIP evaluation questions. The remaining 19 participants did not answer any questions from this section of the survey and their data will not be presented here. Of the 38 respondents, most were IPC consultants (Table 2). Participants mainly came from rural (*n* = 20, 54%) and regional (*n* = 14, 37.8%) areas. Survey results are described in Supplementary material 1.

**Table 2:** Demographics of participants from Residential Aged Care Facilities who participated in the survey

Position	*n* (%)
Aged care IPC lead	10 (26.3)
IPC consultant	19 (50)
Nurse unit or site manager	3 (7.9)
Pharmacist	1 (2.6)
Executive manager	2 (5.3)
Other	3 (7.9)

IPC = Infection prevention and control

### Description of the VICNISS ACIIP

The ACIIP is divided into three categories: ‘significant organism infections’, ‘staff vaccination’ and ‘resident vaccination’ (Table 3).

**Table 3:** Modules in the VICNISS Aged Care Infection Indicator Program Modules for measuring infections and vaccinations in Residential Aged Care Facilities

Category	Module	Denominator	Numerator	Timeframe
Significant organism infections	MRSA, VRE, *C.difficile*	All resident occupied bed days	All residents with MRSA, VRE or *C.difficile* infection	Continuous between January and December each year
Staff vaccination	Influenza	All staff (optionally volunteers) that worked one or more shifts during the specified timeframe	Vaccination status of staff and optionally volunteers	Continuous between April and August each year
Resident vaccination	Herpes zoster	1. All residents 2. Residents aged 70–79 years old.	Vaccination status of residents present on the survey day	Single day in May or September each year
	Influenza	All residents		
	Pneumococcal	Residents aged > 70 years only		

VICNISS = Victorian Healthcare Associated Infection Surveillance System, MRSA = Methicillin resistant *Staphylococcus aureus*, VRE = Vancomycin resistant enterococci, *C. difficile* = *Clostridioides difficile*

**Fig. 1:**
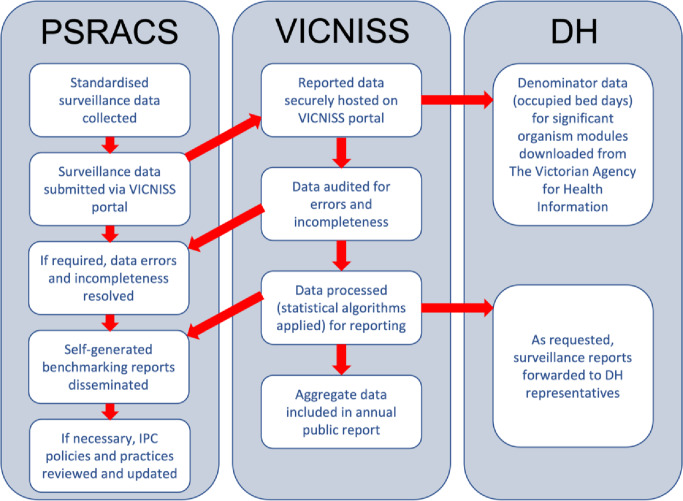
The structure of the VICNISS Aged Care Infection Indicator Program as it relates to data organisation and sharing between services and government

A simple representation of the VICNISS ACIIP is provided in Fig. 1. At the PSRACS, data are collected and submitted online via the password secure VICNISS portal predominately by Infection Control Consultants. Denominator data for the significant organism infection modules is downloaded from the Victorian Agency for Health Information (VAHI) [11]. Once all data are submitted, PSRACS are able to access and compare their data against previous and state-wide aggregate reports. At VICNISS, one Operations Director, two clinical (IPC consultants) and two IT personnel alongside other duties are primarily responsible for co-ordinating the VICNISS ACIIP.

PSRACS = Public Sector Residential Aged Care Services; VICNISS = Victorian Healthcare Associated Infection Surveillance System; DH = Department of Health; IPC = Infection Prevention and Control.

### ACIIP attributes

ACIIP is described below in accordance with the CDC evaluation attributes. Key quotes from VICNISS staff members are provided in Table 4. The quotes are not specifically attributed to participants to maintain anonymity given the small sample size, in accordance with ethical approval for this study.

**Table 4:** Key quotes from VICNISS staff recorded from semi-structured interviews mapped to each CDC evaluation attribute

CDC attribute	Key quotes
**Simplicity**	*“[usability is] something that I am always encouraging that IT staff, it’s like “okay, stop, let’s have a look, how would a user find that? Is it going to be simple for them? Is it clean?”… so simplicity is key… And the other thing is that we do have staff … that advocates very well for the staff in aged care … I think we do produce a good product that is simple for people to use.” (VICNISS staff member)* *“It can be difficult to get pathology reports sometimes… results can go to the GP clinic and we don’t see them at all.” (IPC Consultant)* *“I think the pneumococcal and HZV data is not helpful. This information is difficult to obtain.” (IPC Lead)* *“We gave them five days a week contact with an infection control nurse but also access to the infectious diseases doctors” (VICNISS staff member)* *“I just think it’s really important to make it, to make your availability known, to encourage them to phone early and … to ask them about anything to do with infection control, but if they has an infection … the likelihood of them having had a recent MRSA infection was small so every time they experienced that process, it was like they were starting from scratch, so I said it was much better for us to go through it as a pair and you know, then you can find your way around it again and we know that we’re getting the correct definition.” (VICNISS staff member)* *“it’s all automated. So we set it up and it runs by itself. We use systems to automate that.” (VICNISS staff member)* *“It was more IT development that [the funding] went towards more than anything because that’s the thing that usually is the kind of the critical factor for this … We have maintained it but there hasn’t been any additional funding…it’s really now part of our routine operations.” (VICNISS staff member)*
**Flexibility**	*“we’re trying to now spread [responsibility] across as we hire new people so that they’ll be a little bit more flexible in their duties, you know we used to have one person to the large hospitals one person did a small hospitals one person did the private hospitals, but as people leaving the staff change we’re trying to build in multiple roles” (VICNISS staff member)* *“we’re trying to build in some succession planning and some redundancy so all of the infection control staff that we employ now, they can all answer questions about basic surveillance” (VICNISS staff member)* *“once you work at VICNISS and you know how to navigate the site … with any module if you get queries your first port of call’s the protocol, and you know every single module’s set up exactly the same. So I suppose it, I know exactly where to look number one to look at the performance indicators and see what they should be doing, and then, if they have any queries about a particular module and you know MRSA, VRE, C. diff, those things are the same across all the hospitals and aged care and flu” (VICNISS staff member)* *“Small changes could be done pretty quickly. What caused an issue for [IT staff] was if they had millions of small changes, and we experienced that during the COVID because they kept wanting more modules more surveillance on different groups, so they, their workload was huge.” (VICNISS staff member)*
**Data quality**	*“the issue is we haven’t done any validation so we haven’t really gone into those homes in much detail to see if that they’re being missed” (VICNISS staff member)* *“180 odd age care homes it’s nearly impossible to do a thorough data validation on each of them. I started to do a random pick of them, and they were they were pretty good, but sometimes you would you would just notice a discrepancy in what they had put in.” (VICNISS staff member)* *“at the end of every quarter we chase them to make sure they’ve submitted [surveillance data] and, from what I can see… they don’t seem to have a lot of infections or events that need to be entered so mainly we’re chasing them to confirm that the zeros are correct” (VICNISS staff member)* *“from what I can see they pretty much don’t submit any of these things [MROs] so whether they really are zeroes, or they just don’t have a good system for finding them or they don’t know how to submit it you know, or maybe they don’t have them, they might not have the infections.” (VICNISS staff member)* *“We rarely have any infections with significant organisms.” (IPC Consultant – survey)* *“there is specific training that’s required, most of the staff… are not necessarily surveillance experts … so they do require training in surveillance, because … If they’ve just had a clinical role in the past surveillance is a little bit different and using surveillance definitions, can be a challenge to some of them” (VICNISS staff member)* *“if someone leaves or they give the role to someone else and we [VICNISS] don’t know that and then the new person sort of slips through without getting official training from us, they just sort of tell them how they’ve been doing it in that way, I guess, bad habits can get picked up one person tells someone who tells someone else and before you now they’ve sort of missed a few of the vital points.” (VICNISS staff member)* *“two-yearly when the when the manual was updated, we would do Zoom education on each of [the infection modules] and again I can’t remember the breakdown of aged care, who attended, but I think it was, it was pretty good” (VICNISS staff member)*
**Acceptability**	*“medical staff engagement in this has been difficult with time pressures for IPC it is difficult to raise awareness and drive [the] program” (IPC Consultant - survey)* *“if they were short staffed or someone was off sick that infection control role was the first to go” (VICNISS staff member)* *“I think, in many instances, the infection control role is not their primary focus because they have to focus - they have so many fingers in so many pies and they’ve got to prioritise” (VICNISS staff member)* *“Surveillance is one thing. Education of staff is an entirely different thing. IPC Consultant doing the data collection has not had time to feedback information and education to staff in RACs.”* (IPC Consultant - survey)
**Representativeness**	*“we came up with a list of indicator items that we thought they could report … and then we piloted it and set it in place for the public sector aged care services and they’ve actually been doing it ever since and those indicators are things that are relatively simple for them to report, like MRO infections … so things like MRSA infections in wounds or wherever they might occur, which is a bit different to our other hospital programs, VRE infections, and they also have to report CPE…. The other things that they report on that we came up with was one more kind of process indicators so things like the vaccination status of the both the residents and the staff” (VICNISS staff member)* *“The C diff module required a little bit more because to adapt the definition suitable to an aged care resident, because their situation is different from acute care and so that involved quite a process … to see what we needed to amend or add.” (VICNISS staff member)*
**Timeliness**	*“you’re not going to get January’s till you know, in early April” (VICNISS staff member)* *“I guess at the end of each quarter you, you’re responsible, you needed to see how each facility was going with regards to submitting or completing their data entry… I would do it virtually every month because I encouraged the IPC leads to in aged care to submit, yeah to do their surveillance plan and if they had a an infection in those three modules to enter that data monthly because it’s usually easier for them memory wise to get that information down then than to go at the end of the quarter when all of a sudden, everything has to be due” (VICNISS staff member)*
**Stability**	*“I think the systems that we have built are core to the program … [it] is designed and developed that that’s then been embedded into custom software that [the] VICNISS IT team have developed.” (VICNISS staff member)* *“I can probably remember two or three times we’ve had an outage but it’s so infrequent. You know, we’re talking over 20 years.” (VICNISS staff member)* *“we back up every hour throughout the day and then we do a full back up every night … the backups are encrypted … And then the authentication is the other part of that so it’s fine to have it sitting on a server but who can access the servers and there’s only a small core of people that can actually access those servers, being the IT staff directly.” (VICNISS staff member)*
**Usefulness**	*“we can sit back and passively collect all this information, which is essentially what we’ve done without any resourcing but we don’t ever or we haven’t to date really attempted to change anything so, for example, the vaccination record, so we’re relying on that age care home to be keeping good records of resident vaccinations and also in the case of the pneumococcal vaccine to even ask that perhaps when the residents are admitted like is that a question that gets asked is that not a question that gets asked you know and how these things are documented, so we have no real sense of how well aged care homes are documenting these things, … we’ve never really tackled … validation and quality improvement” (VICNISS staff member)* *“it needs to be regularly assessed to see if a module is worthwhile continuing because, like the VRE, they hardly do get any VRE, so why are we asking them to do it? I know it’s only a matter of saying zero, zero, zero, but I don’t know what the worth of the data is if it’s just constantly zero zero.” (VICNISS staff member)* *“you know I just wonder how helpful, it is in its current form… I mean I suppose it just gets them involved and make sure someone’s keeping an eye on things and, so you know there’s possibly other modules they could do or other things they could do” (VICNISS staff member)*

### Simplicity

ACIIP uses a simple data structure, requiring participants to enter data into a single form for each module (Table 3). VICNISS staff were conscious of making ACIIP easy to use for PSRACS staff. Most survey participants agreed or strongly agreed that entering significant organism data into the ACIIP system is simple (*n* = 35, 92.1%). However, some survey respondents said that finding the data to enter could be difficult. The ACIIP portal contains protocols which outline what data is required in each module and an example of the form to be filled out.

VICNISS staff differed in their view of user simplicity, saying that PSRACS staff can have difficulty with using the ACIIP portal because of infrequent use and can often forget how to use the portal and complete their submission. VICNISS staff sought to reduce these impacts by being available for PSRACS staff to phone and ask questions, and by providing expertise in IPC and infectious diseases.

VICNISS staff explained and document analysis showed that ACIIP does not require a large amount of resources to be maintained, and storing and backing up of data is automated. Initial funding for the program largely went towards IT development, but it is now part of routine operations as the program has not received additional funding.

### Flexibility

ACIIP has demonstrated its flexibility in that it can be updated as required when there are changes to guidelines or definitions for infections. One VICNISS staff member explained that this required coordination between IT staff to update the webforms and IPC staff to update the protocols.

VICNISS IPC staff were previously siloed in their areas of expertise, with one saying *“we used to have one person do the large hospitals, one person did the small hospitals, one person did the private hospitals”*. However, with changing personnel, VICNISS is aiming to have IPC staff who are flexible in their knowledge and duties to ensure there is redundancy in the system.

### Data Quality

Interview participants noted that it can be difficult to check the validity of the data submitted to ACIIP. MRO modules submitted by PSRACS staff showed no cases of VRE, MRSA or *Clostridioides difficile* in 2021 or 2022. VICNISS staff stated that they often had to follow up with facilities that entered zero infections to confirm they were correct, with some expressing concern that infections might be missed. Comments from RACF staff in the survey indicated that these results are correct, stating that they *“rarely have any infections with significant organisms”* (IPC Consultant).

VICNISS provides specific training for new staff participating in the ACIIP, citing the importance of education for staff who are trained in clinical roles and are unfamiliar with surveillance activities. However, with frequent staff change overs, not all RACF staff are captured for training, with only half of PSRACS staff surveyed reporting that they had attended an online training session for the significant organism module (*n* = 19, 50%).

The definitions used for the significant organism modules were developed based on the 2002 Australian Infection Control Association surveillance definitions for multi-resistant organisms [12]. These definitions were not validated before their use in the ACIIP.

### Acceptability

Participation in ACIIP is required for all Victorian PSRACS. It is rare that a facility will not complete data entry.

Survey participants understood the value of participating in ACIIP, with all agreeing or strongly agreeing that reporting of significant organisms and staff vaccination compliance is of public health importance (n = 38, 100%). However, one survey participant noted issues in engaging medical staff in vaccination surveillance, saying *“with time pressures for IPC it is difficult to raise awareness and drive [the] program”* (IPC Consultant).

VICNISS staff noted that surveillance was often not a priority, saying that the *“infection control role was the first to go”* when facilities were short staffed, and that workloads for IPC staff increased considerably when surveillance activities needed to be done.

### Representativeness

The ACIIP was developed specifically for the aged care sector and was designed to include indicators that are important for RACFs to be aware of and are simple to collect.

Victorian PSRACS are required to participate in the ACIIP and as such, all of these facilities will typically submit data.

### Timeliness

PSRACS are required to submit a surveillance plan at the end of the calendar year. Document analysis and staff interviews showed that VICNISS staff often need to follow up with facilities to ensure these are submitted; this can take approximately two weeks.

Significant organism data (Table 3) are submitted by PSRACS every quarter. Facilities are given four weeks from the end of the quarter to complete data submission, which allows time for them to receive any outstanding pathology reports required for entering organism data. VICNISS staff reported that most facilities will typically submit their data following this reminder email. The few facilities that do not submit by this time are contacted by a VICNISS staff member, with VICNISS staff saying that these follow ups can also take approximately two weeks.

ACIIP users are given a timeline of submission dates for each module a year in advance, to allow time for planning. Reports of these results are returned to the facilities approximately one month after the submission due date.

Almost all survey participants agreed (*n* = 21, 55.3%) or strongly agreed (*n* = 16, 42.1%) that the time required to participate in the significant organism modules is justifiable.

### Stability

ACIIP uses custom software that was developed by the in-house IT team. A VICNISS staff member stated that the system is “*very stable… very robust”* and is encrypted and regularly backed up. Unscheduled outages and failures are rare. Confidentiality of participating facilities is maintained.

### Usefulness

Victorian PSRACS are required to provide data for a number of performance indicators. The modules included in the ACIIP address several of these, and the data is provided to the Victorian Department of Health. At a facility level, RACFs are able to view a report of their own data and can compare to data submitted in previous years. Quarterly and annual reports that provide aggregate data from all Victorian PSRACS allows participants to understand their performance in the context of all other participating facilities.

VICNISS staff questioned the benefit of capturing data on significant organisms given that the results were frequently zero. In contrast, survey participants also agreed that participating in the significant organism surveillance (*n* = 36, 94.7%) and vaccination surveillance (*n* = 35, 94.6%) modules was useful for their facility. All survey participants also agreed (*n* = 15, 39.5%) or strongly agreed (*n* = 23, 60.5%) that surveillance of significant organism infections in RACFs is of public health importance. However, fewer agreed that participation had led to initiation of IPC interventions in their facilities (significant organism module: *n* = 20, 52.6%; vaccination module: *n* = 23, 62.2%).

Those who had made changes said the vaccination module had led to *“updating of admission forms to ensure past vaccination data is captured”* (IPC consultant), *“more robust policies and data keeping”* (IPC consultant) and that it *“Helps to identify who needs to be vaccinated after entering aged care. Enables good tracking of staff vaccinations also.”* (IPC Lead). Significant organism infection modules provided *“useful information for education sessions”* (IPC Consultant).

These improvements were driven by RACFs, with one VICNISS staff member stating that facilities frequently don’t have the resources for quality improvement initiatives.

## Discussion

ACIIP is a unique infection surveillance program in Australia. It provides important data for understanding performance and initiating improvement activities in RACFs. This study is the first time the program has been evaluated. Our evaluation provides important implications for understanding and improving infection surveillance in the aged care setting in Australia and other countries. The results of this evaluation will also provide important recommendations for the development of NISPAC.

Several strengths and weaknesses of the ACIIP are identified in the current evaluation. The system benefits from a multidisciplinary team of experts who support RACF staff to correctly use ACIIP. However, the follow-up required by staff to educate users can be time consuming. While the ACIIP portal contains protocols for users on what data is required, there is little information on how to enter data into the portal and how to navigate it. Personal contact from experts has been shown to encourage enrolment and participation in a national surveillance program [13]. However, this requires extensive resourcing and a large number of personnel who can be deployed nationally. At present, it is unlikely that NISPAC would have the resources to provide this level of support. Improved digital reference materials and protocols that demonstrate to users not only what data needs to be entered, but how and where to enter it should be considered in the development of NISPAC. These materials would allow users to undertake self-guided learning, reducing the burden on VICNISS staff.

Similarly, VICNISS staff are currently required to follow-up completion of modules with users. While most users do submit data in a timely manner, with email contact successfully reminding others to submit, phone calls to the remaining facilities can be time consuming for VICNISS staff. It is likely that with implementation of NISPAC, many more facilities would need to be followed up to complete surveillance, which would require far more resources and staff time. Email reminders which provide information on how to submit data may be sufficient to encourage submission of data at a national level, but this is an important consideration for staff resourcing required for NISPAC [14].

The quality of surveillance data submitted to ACIIP is difficult to assess and VICNISS staff raised concerns about data validity. Evaluation of a surveillance system in Norway similarly demonstrated the difficulties with validating surveillance data [14]. This reinforces the need to have high quality protocols and education materials for users, and also highlights the importance of having access to expert staff who can guide users on how to collect data. That most participants reported that identifying significant organisms for surveillance was simple provides a level of assurance that users are confident in the data they are entering into the system.

VICNISS IT staff have developed a robust system which can be easily adapted to changes and has proved its stability. ACIIP benefits from this dedicated team of experts who are able to quickly make changes and improvements to the system as needed. Expanding the program nationally as part of NISPAC will mean the IT system will need to support data from approximately 2,600 facilities rather than the current 179 [15]. It is unclear how well the system will be able to handle this amount of data, as well as the operational activities required to manage the considerable increase in users. More testing of the IT system’s capacity will be needed during the development of NISPAC.

ACIIP benefits facilities by increasing awareness of significant organism infections and vaccinations amongst staff. Evaluation of a similar program in the US also found that awareness of IPC was improved simply by participating in infection reporting. [13] In contrast, fewer facilities reported initiating quality improvements based on participation in the modules. It is possible that this is due to a lack of resources or funding, but increased support from VICNISS in understanding reports and how to utilise surveillance data could be valuable for RACFs.

There were some limitations to this study. The survey was comprised of several sections with the ACIIP evaluation section placed at the end. The survey may have been too long for RACF staff to complete, and as such, some participants who were eligible to complete the ACIIP evaluation section did not reach it, which reduced the possible sample size for the quantitative analysis. The general concordance between the VICNISS staff interviews and the survey data indicates that the results received were still representative of the target population. For points where there was discordance between VICNISS staff and PSRACS staff, further qualitative analysis with PSRACS staff may be required in future to delve into the reasons for this, however, it is unlikely to change the overall understandings of ACIIP gained from this study. Participation in the survey was anonymous, so it was not possible to check for duplication. However, there were no consistent demographics results across participants, so it is unlikely that duplication occurred for this cohort.

## Conclusion

ACIIP is useful for RACFs to report rates of significant organism infections and vaccination compliance. Staff appreciate the importance of collecting this data and the benefits it has had on improving awareness of IPC activities in their facilities. This evaluation has highlighted areas for consideration to improve the scalability of the program to a national level – namely, data validation, IT system capacity and need for user education/support.

### Electronic supplementary material

Below is the link to the electronic supplementary material.

**Supplementary Material 1:** Victorian PSRACS staff survey results

**Supplementary Material 2:** COREQ checklist for study conduct

## Data Availability

The datasets generated and analysed during the current study are not publicly available to maintain privacy of individuals. The survey data are available from the corresponding author only on reasonable request.

## List of abbreviations

ACIIP: Aged Care Infection Indicator Program
IPC: Infection Prevention and Control
NCAS: National Centre for Antimicrobial Stewardship
PSRACS: Public Sector Residential Aged Care Services
RACF: Residential Aged Care Facility
VICNISS: Victorian Healthcare Associated Infection Surveillance System
