# Supplementary material for: Evaluation of an Infection surveillance program in residential aged care facilities in Victoria, Australia
Source: BMC Public Health. 2024 Jan 22;24:254. doi: 10.1186/s12889-023-17482-x (PMC10801934; doi:10.1186/s12889-023-17482-x)
Supplement: Supplementary file 1 — Supplementary Material 1: Victorian PSRACS staff survey results [file 12889_2023_17482_MOESM1_ESM.docx]

**Supplementary Material 1 – Survey results**

**Part 1 – Infection indicator program, n (%)**

|  | Aged Care IPC Lead | Registered Nurse | IPC Consultant | Unknown | Other | Total |
| --- | --- | --- | --- | --- | --- | --- |
| Who primarily collects and submits the significant organism infection surveillance data? | 8 (22.86) | 1 (2.86) | 22 (62.86) | 1 (2.86) | 3 (8.57) | 35 |

| **CDC Guidelines attribute** | **Question** | **Strongly disagree** | **Disagree** | **Unsure** | **Agree** | **Strongly agree** | **Total** |
| --- | --- | --- | --- | --- | --- | --- | --- |
| Acceptability | The reporting of significant organism infections in RACS is of public health importance | 0 (0) | 0 (0) | 0 (0) | 15 (39.47) | 23 (60.53) | 38 |
| Timeliness | The time required to participate in the modules is justifiable because of the value of the information collected | 0 (0) | 1 (2.63) | 0 (0) | 21 (55.26) | 16 (42.11) | 38 |
| Simplicity | The process in our RACS for identifying significant organism infection is simple | 0 (0) | 4 (10.53) | 4 (10.53) | 22 (57.89) | 8 (21.05) | 38 |
| Simplicity | The online processes for significant organism infection data submission to VICNISS is simple | 0 (0) | 0 (0) | 3 (7.89) | 26 (68.42) | 9 (23.68) | 38 |

| **CDC Guidelines attribute** | **Question** | **No** | **Yes** | **Total** |
| --- | --- | --- | --- | --- |
| Data quality | Have you attended an online training session about how to participate in the significant organism infection module? | 19 (50.00) | 19 (50.00) | 38 |
| Usefulness | Do you think participation in the significant organism infection surveillance modules is useful for your RACS? | 2 (5.26) | 36 (94.74) | 38 |
| Usefulness | Has participation in the modules helped raise awareness about these infection types in your RACS? | 8 (21.05) | 30 (78.95) | 38 |
| Usefulness | Has participation in the modules led to the initiation of IPC interventions in your RACS? | 18 (47.37) | 20 (52.63) | 38 |

**Part 2 – Vaccination modules, n (%)**

|  | Aged Care IPC Lead | Registered Nurse | IPC Consultant | Unknown | Other | Total |
| --- | --- | --- | --- | --- | --- | --- |
| Who primarily collects and submits staff vaccination data? | 6 (16.22) | 1 (2.70) | 22 (59.46) | 1 (2.70) | 7 (18.92) | 37 |
| Who primarily collects and submits resident vaccination data? | 10 (27.03) | 4 (10.81) | 17 (45.95) | 0 (0) | 6 (16.22) | 37 |

| **CDC Guidelines attribute** | **Question** | **Strongly disagree** | **Disagree** | **Unsure** | **Agree** | **Strongly agree** | **Total** |
| --- | --- | --- | --- | --- | --- | --- | --- |
| Acceptability | The reporting of staff vaccination compliance is of public health importance | 0 (0) | 0 (0) | 0 (0) | 14 (38.89) | 22 (61.11) | 36 |
| Acceptability | The reporting of resident vaccination compliance is of public health importance | 0 (0) | 0 (0) | 2 (5.41) | 13 (35.14) | 22 (59.46) | 37 |

| **CDC Guideline attribute** | **Question** | **No** | **Yes** | **Total** |
| --- | --- | --- | --- | --- |
| Usefulness | Do you think participation in the vaccination surveillance modules is useful for your RACS? | 2 (5.41) | 35 (94.59) | 37 |
| Usefulness | Has participation in the vaccination modules helped raise awareness about these infection types in your RACS? | 6 (16.22) | 31 (83.78) | 37 |
| Usefulness | Has participation in the vaccination modules led to the initiation of IPC interventions in your RACS? | 14 (37.84) | 23 (62.16) | 37 |
