# Supplementary material for: Evaluation of an Infection surveillance program in residential aged care facilities in Victoria, Australia
Source: BMC Public Health. 2024 Jan 22;24:254. doi: 10.1186/s12889-023-17482-x (PMC10801934; doi:10.1186/s12889-023-17482-x)
Supplement: Supplementary file 2 — Supplementary Material 2: COREQ checklist for study conduct [file 12889_2023_17482_MOESM2_ESM.docx]

**COREQ CHECKLIST**

| **Domain 1: Research team and reflexivity** |  |  |
| --- | --- | --- |
| *Personal Characteristics* |  |  |
| 1. Interviewer/facilitator | Which author/s conducted the interview or focus group? | EW |
| 2. Credentials | What were the researcher’s credentials? E.g. PhD, MD | EW: MPH  AR: PhD  DD: PhD  LW: PhD  NB: PhD |
| 3. Occupation | What was their occupation at the time of the study? | Infection prevention and control consultant, clinicians and researchers. Ranging from project officer to Associate Professor and clinicians. |
| 4. Gender | Was the researcher male or female? | The interviewer is female. The research team are both male and female. |
| 5. Experience and training | What experience or training did the researcher have? | Researchers have expertise in qualitative methodologies including conducting interviews and thematic and content analysis. |
| *Relationship with participants* |  |  |
| 6. Relationship established | Was a relationship established prior to study commencement? | Yes. The interviewer is a colleague of the interviewees, but at the time of participation, had not worked directly with the participants and had had little previous contact. |
| 7. Participant knowledge of the interviewer | What did the participants know about the researcher? e.g. personal goals, reasons for doing the research | Participants were provided with information about the reasons for conducting the research. The participants knew the role of the researcher in the project. |
| 8. Interviewer characteristics | What characteristics were reported about the interviewer/facilitator? e.g. Bias, assumptions, reasons and interests in the research topic | None. |
| **Domain 2: study design** |  |  |
| *Theoretical framework* |  |  |
| 9. Methodological orientation and Theory | What methodological orientation was stated to underpin the study? e.g. grounded theory, discourse analysis, ethnography, phenomenology, content analysis | Content analysis. |
| *Participant selection* |  |  |
| 10. Sampling | How were participants selected? e.g. purposive, convenience, consecutive, snowball | Purposive. |
| 11. Method of approach | How were participants approached? e.g. face-to-face, telephone, mail, email | Via Zoom. |
| 12. Sample size | How many participants were in the study? | Four. |
| 13. Non-participation | How many people refused to participate or dropped out? Reasons? | All participants who were approached agreed to participate. |
| *Setting* |  |  |
| 14. Setting of data collection | Where was the data collected? e.g. home, clinic, workplace | Via Zoom. |
| 15. Presence of non-participants | Was anyone else present besides the participants and researchers? | No. |
| 16. Description of sample | What are the important characteristics of the sample? e.g. demographic data, date | The participants were staff from VICNISS who are involved in the ACIIP. |
| *Data collection* |  |  |
| 17. Interview guide | Were questions, prompts, guides provided by the authors? Was it pilot tested? | Interview questions were provided by the authors. They were not pilot tested. |
| 18. Repeat interviews | Were repeat interviews carried out? If yes, how many? | No. |
| 19. Audio/visual recording | Did the research use audio or visual recording to collect the data? | Yes, the interviews were audio recorded. |
| 20. Field notes | Were field notes made during and/or after the interview or focus group? | No. |
| 21. Duration | What was the duration of the interviews or focus group? | Interviews ranged from 14-37 minutes. |
| 22. Data saturation | Was data saturation discussed? | Data saturation was not discussed. Saturation was not likely to be reached from these interviews because each staff member interviewed was involved in a different aspect of the ACIIP. This was expected and the data was supplemented with quantitative data and document analysis. |
| 23. Transcripts returned | Were transcripts returned to participants for comment and/or correction? | No. |
| **Domain 3: analysis and findings** |  |  |
| *Data analysis* |  |  |
| 24. Number of data coders | How many data coders coded the data? | Two. |
| 25. Description of the coding tree | Did authors provide a description of the coding tree? | No. |
| 26. Derivation of themes | Were themes identified in advance or derived from the data? | Themes were identified in advance from the CDC Updated Guidelines for Evaluating Public Health Surveillance Systems |
| 27. Software | What software, if applicable, was used to manage the data? | NVivo. |
| 28. Participant checking | Did participants provide feedback on the findings? | No. |
| *Reporting* |  |  |
| 29. Quotations presented | Were participant quotations presented to illustrate the themes / findings? Was each quotation identified? e.g. participant number | Quotes were presented but not identified due to the small sample size, to protect anonymity of the participants. |
| 30. Data and findings consistent | Was there consistency between the data presented and the findings? | Yes. |
| 31. Clarity of major themes | Were major themes clearly presented in the findings? | Yes. |
| 32. Clarity of minor themes | Is there a description of diverse cases or discussion of minor themes? | Only major themes were drawn from the data due to the limited sample size. |
